# PIM kinases mediate resistance to cisplatin chemotherapy in hepatoblastoma

**DOI:** 10.1038/s41598-021-85289-0

**Published:** 2021-03-16

**Authors:** Raoud Marayati, Laura L. Stafman, Adele P. Williams, Laura V. Bownes, Colin H. Quinn, Jamie M. Aye, Jerry E. Stewart, Karina J. Yoon, Joshua C. Anderson, Christopher D. Willey, Elizabeth A. Beierle

**Affiliations:** 1 Department of Surgery, University of Alabama at Birmingham, Birmingham, AL 35233 USA; 2 Department of Pediatric Hematology Oncology, University of Alabama at Birmingham, Birmingham, AL 35233 USA; 3 Department of Pharmacology and Toxicology, University of Alabama at Birmingham, Birmingham, AL 35233 USA; 4 Department of Radiation Oncology, University of Alabama at Birmingham, Birmingham, AL 35233 USA; 5 Department of Surgery, University of Alabama at Birmingham, 1600 7th Ave. South, Lowder Building, Suite 300, Birmingham, AL 35233 USA

**Keywords:** Paediatric cancer, Cancer therapeutic resistance, Cancer stem cells

## Abstract

Despite increasing incidence, treatment for hepatoblastoma has not changed significantly over the past 20 years. Chemotherapeutic strategies continue to rely on cisplatin, as it remains the most active single agent against hepatoblastoma. However, chemoresistance remains a significant challenge with 54–80% of patients developing resistance to chemotherapy after 4–5 cycles of treatment. Stem cell-like cancer cells (SCLCCs) are a subset of cells thought to play a role in chemoresistance and disease recurrence. We have previously demonstrated that Proviral Integration site for Moloney murine leukemia virus (PIM) kinases, specifically PIM3, play a role in hepatoblastoma cell proliferation and tumor growth and maintain the SCLCC phenotype. Here, we describe the development of a cisplatin-resistant hepatoblastoma xenograft model of the human HuH6 cell line and a patient-derived xenograft, COA67. We provide evidence that these cisplatin-resistant cells are enriched for SCLCCs and express PIM3 at higher levels than cisplatin-naïve cells. We demonstrate that PIM inhibition with AZD1208 sensitizes cisplatin-resistant hepatoblastoma cells to cisplatin, enhances cisplatin-mediated apoptosis, and decreases the SCLCC phenotype seen with cisplatin resistance. Together, these findings indicate that PIM inhibition may be a promising adjunct in the treatment of hepatoblastoma to effectively target SCLCCs and potentially decrease chemoresistance and subsequent disease relapse.

## Introduction

Hepatoblastoma is the most common primary liver malignancy in children with a predilection for children younger than 5 years old^1,2^. The incidence of hepatoblastoma in the United States (US) has increased over time with a fourfold increase from 1975 to 2007 when data are extrapolated to the US population^3^. Despite this increase, chemotherapeutic treatment has not changed significantly in the past 20 years, relying primarily on cisplatin as it remains the most active single agent against hepatoblastoma^4,5^. Chemoresistance is a significant challenge in hepatoblastoma as 54–80% of patients develop resistance to chemotherapy after four to five cycles of treatment^6–8^. Children with advanced stage, metastasis, and recurrent disease represent a patient population with limited research opportunities and a dearth of effective treatments making the development of novel and innovative therapeutics imperative.

Stem cell-like cancer cells (SCLCCs) are a subset of cancer cells thought to play a role in metastasis, relapse, recurrence, and chemoresistance^9–12^. They are identified by their ability to (i) form spheres in non-adherent serum-free conditions, (ii) form tumors in immune-deficient mice at low cell numbers, and (iii) express certain cell surface markers^13,14^. We have previously shown that CD133-enriched cells form spheres more readily at lower cell concentrations and form tumors in animals at low cell numbers more frequently than CD133-depleted cells, verifying the utility of CD133 as one such marker for SCLCCs in hepatoblastoma^15^. Multiple studies in a variety of cancer types have implicated SCLCCs in chemoresistance against a variety of chemotherapeutics^16^, and cisplatin, specifically, has been demonstrated to enrich the SCLCC subpopulation^17–19^. Targeting SCLCCs affords a potential mechanism to overcome chemoresistance.

Proviral Integration in Moloney murine leukemia virus (PIM) kinases are a family of serine-threonine kinases that play a role in hepatoblastoma tumorigenicity^20^. We have previously shown that inhibition of PIM kinases with the small molecule AZD1208 decreased CD133 expression and formation of hepatoblastoma tumorspheres^15^. In addition, PIM inhibition combined with cisplatin synergistically decreased tumor growth in vivo leading us to believe that PIM3 plays a role in mediating chemoresistance to cisplatin hepatoblastoma by maintaining the SCLCC phenotype. In the current study, we developed cisplatin-resistant hepatoblastoma xenograft models and showed that cisplatin-resistant cells are enriched for SCLCCs and have higher PIM3 expression. We also demonstrated that PIM inhibition increased sensitivity to cisplatin in the cisplatin-resistant cells, significantly inhibiting proliferation, enhancing cisplatin-induced apoptosis, and reducing the SCLCC phenotype that contributes to cisplatin resistance. These data provide evidence for the role of PIM kinases in chemoresistance in hepatoblastoma and propose PIM inhibition as a strategy to potentially overcome cisplatin resistance by targeting the SCLCC phenotype.

## Results

### Establishment of a hepatoblastoma cisplatin-resistant model

**Figure 1:**
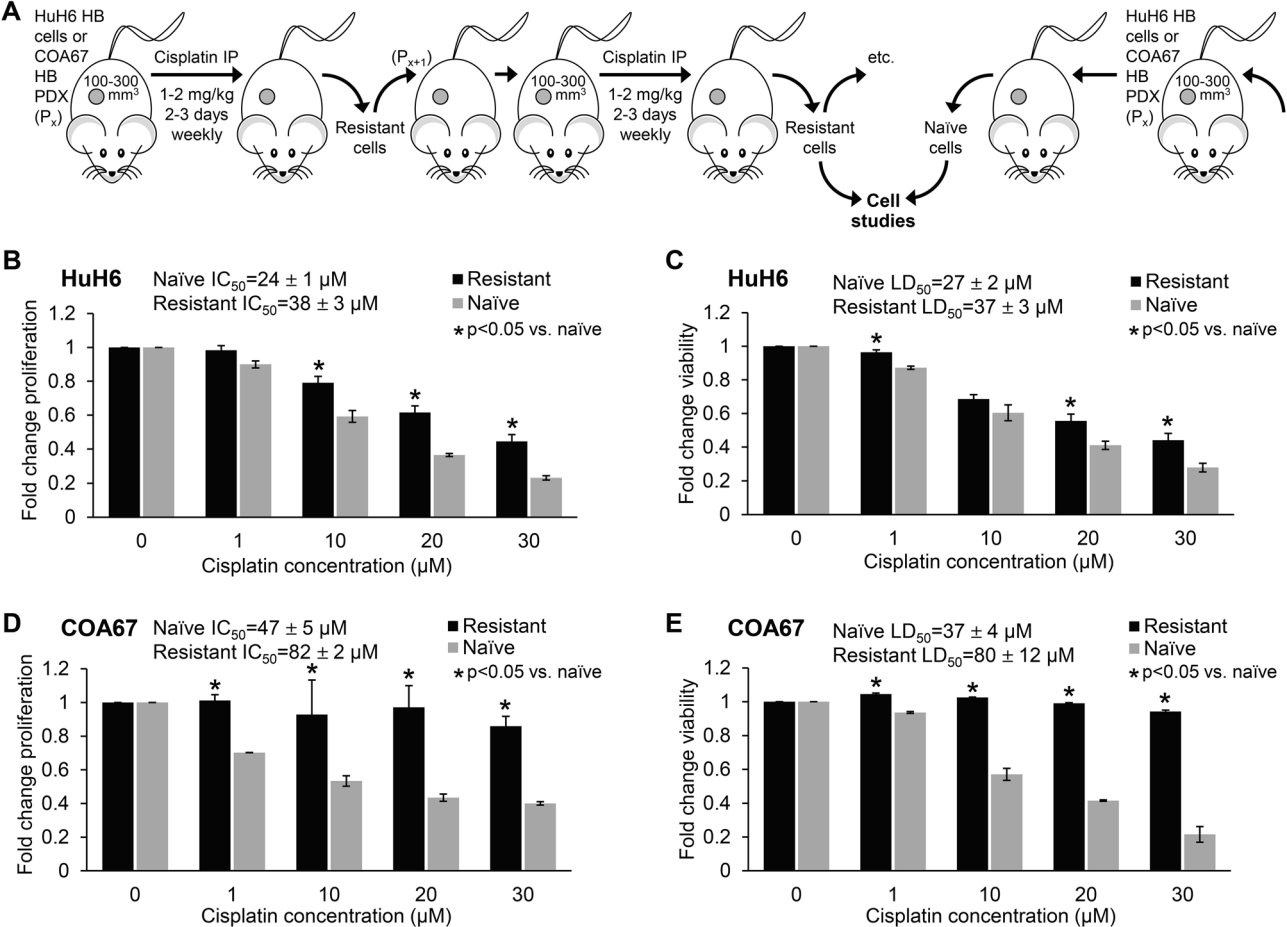
Development of a hepatoblastoma cisplatin-resistant xenograft model. (**A**) Human hepatoblastoma cells, HuH6, or hepatoblastoma patient-derived xenografts, COA67, were injected or implanted, subcutaneously, into the flank of one female athymic nude mouse each. Once tumor volume reached 100 mm^3^ for HuH6 and 300 mm^3^ for COA67, the animal was treated with cisplatin via intraperitoneal injection (2 mg/kg/day for 3 consecutive days weekly for HuH6 and 1 mg/kg/day for 2 consecutive days weekly for COA67). Tumors were harvested to yield “cisplatin-resistant” cells, which were used for studies, and serially passed into a second mouse. Alternatively, tumors from mice not treated with cisplatin were utilized as controls, yielding “cisplatin-naïve” HuH6 and COA67 cells. (**B**–**E**) Cisplatin-resistant and cisplatin-naïve cells were treated with cisplatin at increasing concentrations (0 to 30 µM) for 72 h, and cell proliferation and viability were measured with CellTiter 96 and alamarBlue assays, respectively. (**B**,**D**) Proliferation and (**C**,**E**) viability was less affected given the same dose of cisplatin in resistant compared to naïve cells. The IC_50_ and LD_50_ values were calculated following treatment with cisplatin. Resistant cells had a higher IC_50_ and higher LD_50_ compared to naïve cells. These findings confirmed the cisplatin resistance model in both HuH6 and COA67 hepatoblastoma xenografts. Data reported as mean ± SEM.

In order to understand whether PIM kinases play a role in chemoresistance, we first sought to develop a cisplatin resistant model of hepatoblastoma. Cisplatin-resistant cells were generated by serial passage of HuH6 and COA67 PDX cells in animals. Cells were injected subcutaneously into the flank of athymic nude mice (Fig. 1A). Once tumor volumes reached 100 and 300 mm^3^ for HuH6 and COA67 cells, respectively, the mice were treated with cisplatin via intraperitoneal injection (2 mg/kg/day on three consecutive days and 1 mg/kg/day on two consecutive days, weekly, for HuH6 and COA67, respectively). Tumors were harvested to yield cells less sensitive to cisplatin and were re-implanted into a second animal. Once cells were sufficiently insensitive to cisplatin they were utilized for experimentation. The process required about six passages. These cells are referred to as “cisplatin-resistant” cells throughout the manuscript for ease of explanation and discussion. HuH6 and COA67 cells from mice bearing tumors that were not treated with cisplatin were utilized as controls and referred to as “cisplatin-naïve” cells (Fig. 1A). Immediately after harvest and dissociation, cells were treated with cisplatin for 72 h and cell viability and proliferation were assessed using alamarBlue or CellTiter 96 assays, respectively.

In the presence of cisplatin, cell proliferation and viability were significantly decreased in the cisplatin-naïve but not in the cisplatin-resistant hepatoblastoma cells. Cisplatin-resistant HuH6 cells required a higher half maximal inhibitory concentration (IC_50_, 38 ± 3 µM vs. 24 ± 1 µM, p ≤ 0.05, Fig. 1B) and a higher median lethal dose (LD_50_, 37 ± 3 µM vs. 27 ± 2 µM, p ≤ 0.05, Fig. 1C) of cisplatin compared to cells from untreated mice (cisplatin-naïve cells). Similarly, cisplatin-resistant COA67 cells had a higher IC_50_ (82 ± 2 µM vs. 47 ± 5 µM, p ≤ 0.05) and a higher LD_50_ (80 ± 12 µM vs. 37 ± 4 µM, p ≤ 0.05) than cisplatin-naïve cells (Fig. 1D,E). Collectively, these results indicated that cisplatin-resistant cells exhibited resistance to cisplatin compared to cisplatin-naïve cells demonstrating the successful establishment of hepatoblastoma cisplatin-resistant cells.

### Cisplatin-resistant hepatoblastoma cells are more stem-like

**Figure 2:**
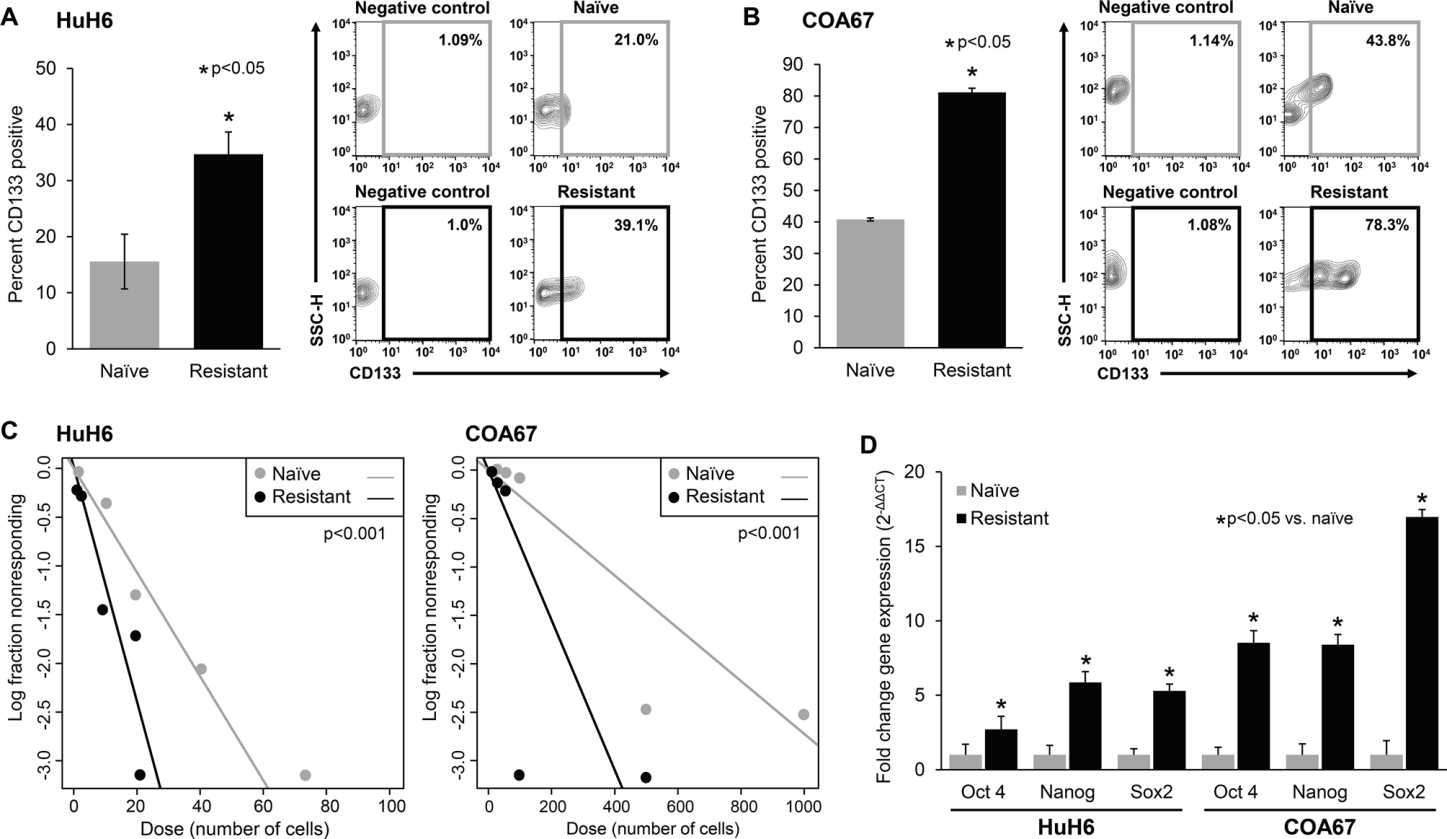
Cisplatin-resistant hepatoblastoma cells have higher frequency of stem cell-like cancer cells (SCLCCs). (**A,B**) CD133 cell surface expression was evaluated by FACS in cells from both cisplatin-naïve and cisplatin-resistant tumors and reported as percent CD133 expression ± SEM. Cisplatin-resistant (**A**) HuH6 and (**B**) COA67 cells had higher expression of CD133. Representative contour plots are shown to the right of the graphs. (**C**) Extreme limiting dilution analysis was performed to evaluate sphere forming ability. Cisplatin-resistant and cisplatin-naïve cells were plated in non-adherent culture conditions at decreasing concentrations of cells (from 5000 to 1 cell per well for HuH6 and 10,000 to 1 cell per well for COA67). After one week, wells were examined by a single blinded researcher and the numbers of wells containing spheres were counted. Sphere forming ability was calculated utilizing the extreme limiting dilution analysis software (http://www.bioinf.wehi.edu.au/software/elda/) and a plot of the log proportion of negative cultures vs. the number of cells plated is shown. The slope of the line is the estimated log-active SCLCC fraction. Cisplatin-resistant cells formed spheres more frequently than cisplatin-naïve cells (p < 0.001), indicating increased stemness. (**D**) Quantitative real-time PCR was utilized to assess the mRNA abundance of Oct4, Nanog, and Sox2. Relative abundance of mRNA was calculated using the ΔΔCt method and reported as mean ± SEM of three biologic replicate experiments. Cisplatin-resistant cells from both HuH6 and COA67 xenografts had significantly increased abundance of all three stem cell markers compared to naïve cells (p < 0.05), further indicating an increase in stemness.

The presence of SCLCCs is hypothesized to be a driver of chemoresistance in cancer. We have previously identified CD133 as a marker for SCLCCs in hepatoblastoma^15^. To evaluate cancer cell stemness, we examined the expression of CD133 cell surface marker, the ability of cells to form tumorspheres, and mRNA abundance of common markers of cell stemness. FACS sorting demonstrated that cisplatin-resistant cells expressed CD133 at a higher frequency than cisplatin-naïve cells (35 ± 4% vs. 16 ± 5% for HuH6 and 81 ± 0.5% vs. 41 ± 1% for COA67 cells, p ≤ 0.05, Fig. 2A,B).

One method to identify SCLCCs is the ability to form spheres in non-adherent serum-free conditions. Using an extreme limiting dilution analysis (ELDA), we found that both cisplatin-resistant HuH6 and COA67 cells formed spheres more frequently and at lower cell concentrations than cisplatin-naïve cells (p < 0.001, Fig. 2C and Supplementary Info Figure S1A–C), indicating an increase in the SCLCC phenotype.

Another known marker of the SCLCC subpopulation is the expression of the three transcription factors, octamer-binding transcription factor 4 (Oct4), homeobox protein Nanog, and sex determining region Y-box 2 (Sox2). Quantitative real-time PCR (qPCR) evaluation comparing mRNA from cisplatin-naïve and cisplatin-resistant hepatoblastoma cells demonstrated a significantly higher mRNA abundance of these SCLCC markers (Fig. 2D, p < 0.05) in the cisplatin-resistant cells, further indicating that resistant cells have a more stem-like phenotype.

Lastly, we examined alternative stem cell markers as well as pathways known to be essential for self-renewal and maintenance of stem cells, such as the Notch pathway. Using qPCR, we found that the abundance of mRNA of the cancer stem cell marker Aldehyde dehydrogenase 1A3 (ALDH1A3) and the Notch pathway genes (specifically NOTCH1 and NOTCH3) was upregulated in the cisplatin-resistant compared to the cisplatin-naïve hepatoblastoma cells (p < 0.05, Supplementary Info Figure S2).

### Cisplatin-resistant hepatoblastoma cells have higher PIM3 activity and expression

**Figure 3:**
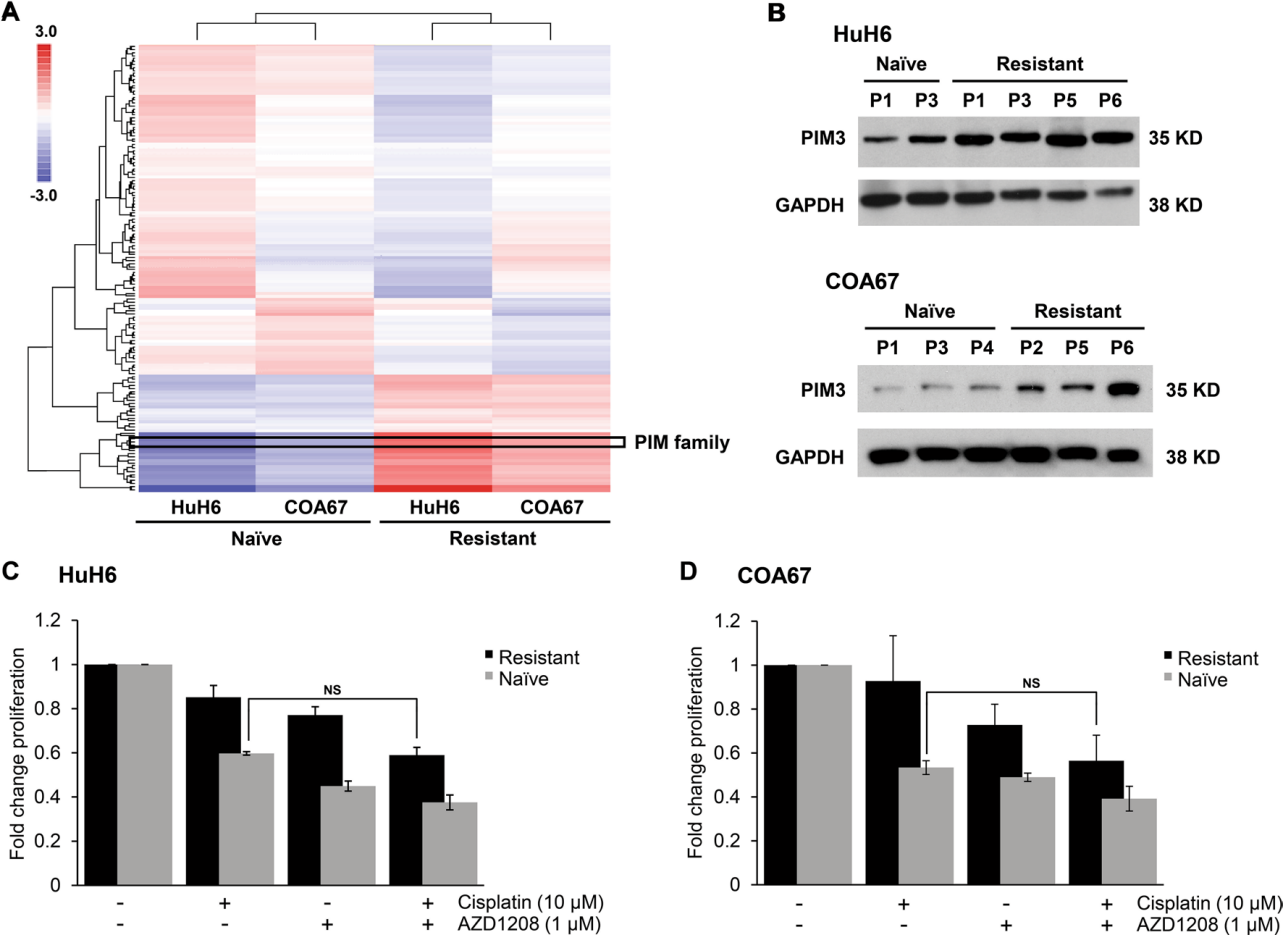
Cisplatin-resistant hepatoblastoma cells have higher activity and expression of PIM3 kinase and PIM inhibition increases their sensitivity to cisplatin. (**A**) Kinomic profiling of HuH6 and COA67 cisplatin-naïve and resistant cells was performed using a PamStation12 microarray. The heatmap displays kinomic signature data with kinomic peptides across all conditions (colored by log difference from mean per cell type, per peptide). Clustering is unsupervised, via a geometric means-distance method, with peptides clustered by row, and samples clustered by column. PIM family kinase targets (*boxed region shown*) were significantly activated in cisplatin-resistant compared to cisplatin-naïve HuH6 and COA67 tumors. (**B**) Immunoblotting was performed on lysates from both HuH6 and COA67 cisplatin-naïve and cisplatin-resistant tumors from various passages (P). PIM3 expression was higher in cisplatin-resistant compared to cisplatin-naïve tumors and increased as cells became more insensitive to cisplatin. GAPDH was used to confirm equal protein loading. (**C**) HuH6 and (**D**) COA67 cisplatin-naïve and resistant cells were treated with 1 µM of AZD1208, 10 µM of cisplatin, or both drugs for 72 h and proliferation evaluated with CellTiter 96 assay. The addition of AZD1208 to cisplatin reduced proliferation of resistant cells to levels comparable to those of naïve cells treated with cisplatin alone, indicating the ability of AZD1208 to sensitize resistant cells to cisplatin. *NS* not significant.

To examine activated kinases that may play a role in hepatoblastoma chemoresistance, we performed a kinome assay on paired cisplatin-resistant and naïve tumors from HuH6 and COA67 xenografts using a whole chip comparative analysis of both Tyrosine chip (PTK) and Serine-Threonine chip (STK) arrays. Unsupervised clustering of kinomic peptides across all conditions clustered by row, with samples clustered by column, resulted in the heatmap shown in Fig. 3A. Notably, the cisplatin-naïve and cisplatin-resistant tumors clustered together rather than with their paired cell-of-origin (HuH6 or COA67) sample, suggesting a robust cisplatin resistance signature. Furthermore, comparison across clustered groups identified target peptides associated with PIM family kinases were highly activated (mean final score > 2.5) in the resistant tumors compared to the cisplatin-naïve tumors.

To validate and corroborate the findings of the kinome assay, immunoblotting was performed for PIM3 expression. Immunoblotting demonstrated increasing PIM3 expression in both HuH6 and COA67 with increasing insensitivity to cisplatin (Fig. 3B), indicating that PIM3 expression correlates with cisplatin resistance in hepatoblastoma.

### PIM inhibition with AZD1208 increases sensitivity of cisplatin-resistant hepatoblastoma cells to cisplatin

Proliferation of cisplatin-resistant HuH6 and COA67 cells was assessed in the presence of both cisplatin and/or the PIM inhibitor, AZD1208. The addition of 1 µM of AZD1208 to cisplatin in both HuH6 and COA67 cisplatin-resistant hepatoblastoma cells resulted in decreased proliferation to levels of cisplatin-naïve cells treated with cisplatin alone (0.59 ± 0.04 fold change proliferation in HuH6 cisplatin-resistant cells treated with cisplatin and AZD1208 vs. 0.60 ± 0.01 fold change proliferation in HuH6 cisplatin-naïve cells, p = 0.38, Fig. 3C, and 0.56 ± 0.12 fold change proliferation in COA67 cisplatin-resistant cells treated with cisplatin and AZD1208 vs. 0.53 ± 0.03 fold change proliferation in COA67 cisplatin-naïve cells, p = 0.43, Fig. 3D), indicating that PIM inhibition with 1 µM of AZD1208 sensitized HuH6 and COA67 cisplatin-resistant cells to cisplatin. Treatment with both AZD1208 and cisplatin significantly decreased proliferation in both HuH6 and COA67 cisplatin-resistant hepatoblastoma cells compared to either drug alone and to untreated controls (p < 0.05, Fig. 3C,D).

### PIM inhibition with AZD1208 promotes cisplatin-induced apoptosis of hepatoblastoma cells

**Figure 4:**
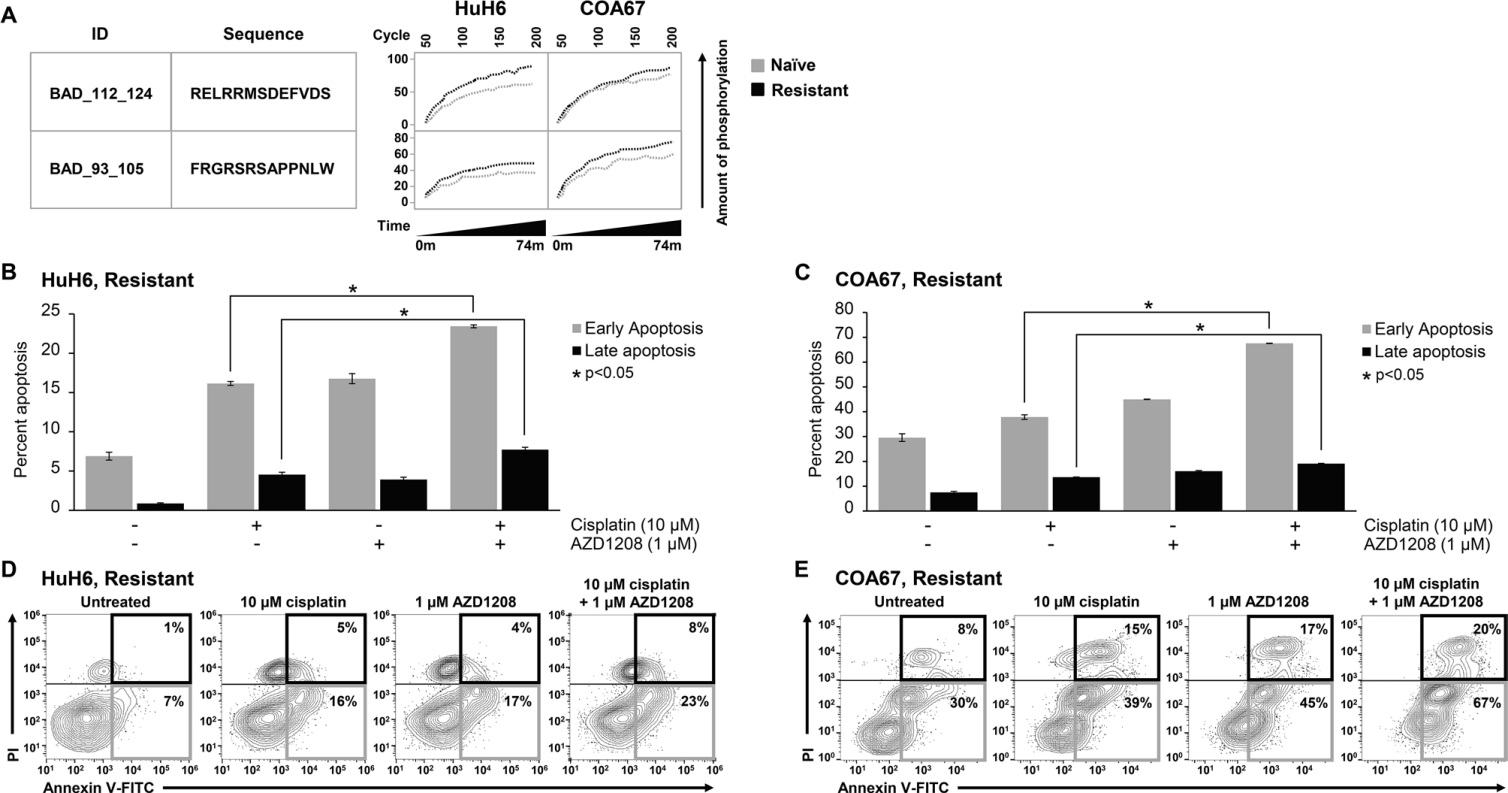
PIM3 inhibition with AZD1208 promotes cisplatin-induced apoptosis. (**A**) Kinetic phosphorylation curves for peptides identified as potential PIM3 targets were overlaid for both cisplatin-naïve and cisplatin-resistant tumors. Phosphorylation of the pro-apoptotic protein BAD at phosphorylation sites that inhibit apoptosis was increased in resistant compared to naïve tumors in both HuH6 and COA67 xenografts, indicating decreased apoptosis in resistant cells. (**B**–**E**) Cisplatin-induced apoptosis was assessed by flow cytometric analysis of Annexin V/PI dual staining. (**B**) HuH6 and (**C**) COA67 cisplatin-resistant cells with or without treatment with 1 µM AZD1208 and/or 10 µM cisplatin for 72 h (for HuH6) and 24 h (for COA67) were stained and analyzed. Values expressed as mean percentage ± SEM. PIM inhibition with AZD1208 significantly promoted early (Annexin V + PI- cells, lower right quadrant (**D**,**E**)) as well as late (Annexin V + PI + cells, upper right quadrant (**D**,**E**)) apoptosis in cisplatin-resistant cells, indicating that the addition of PIM inhibition enhanced cisplatin-mediated apoptosis. Representative contour plots shown for both (**D**) HuH6 and (**E**) COA67 cisplatin-resistant cells along with appropriate staining controls (top panels).

Most chemotherapeutic drugs exert their anti-cancer activity by inducing apoptosis^21^. Thus, resistance to apoptosis may constitute an important factor in limiting the effectiveness of chemotherapy and conferring drug resistance^22,23^. We have previously shown that PIM kinases regulate the pro-apoptotic protein Bad in hepatoblastoma^20^. In analyzing the previously described kinome data, we found that the kinetic phosphorylation of a Bad substrate (at serine 93 and 112) was increased in the cisplatin-resistant versus naïve tumors (Fig. 4A). Given that phosphorylation of Bad at these residues inactivates the protein’s ability to induce apoptosis, and that treatment with AZD1208 increased sensitivity of cisplatin-resistant cells to cisplatin, we sought to determine if AZD1208 would also sensitize cisplatin-resistant cells to cisplatin-induced apoptosis.

Flow cytometry analysis of Annexin V/PI dual stained cisplatin-resistant cells demonstrated that PIM inhibition with AZD1208 significantly promoted early (shown by the accumulation of Annexin V + PI− cells, Fig. 4D,E, *lower right quadrant, grey boxes*) as well as late (Annexin V + PI + cells, Fig. 4D,E, *upper right quadrant, black boxes*) apoptosis in cisplatin-resistant cells HuH6 (Fig. 4B) and COA67 (Fig. 4C) compared to either AZD1208 or cisplatin alone, indicating that the addition of AZD1208 enhanced cisplatin-mediated apoptosis. Representative contour plots are shown for both HuH6 (Fig. 4D) and COA67 (Fig. 4E) cisplatin-resistant cells. Representative contour plots of staining controls are supplied in Supplementary Info Figure S3.

### PIM inhibition with AZD1208 reduces the stem cell-like cancer cell (SCLCC) phenotype seen with cisplatin resistance

**Figure 5:**
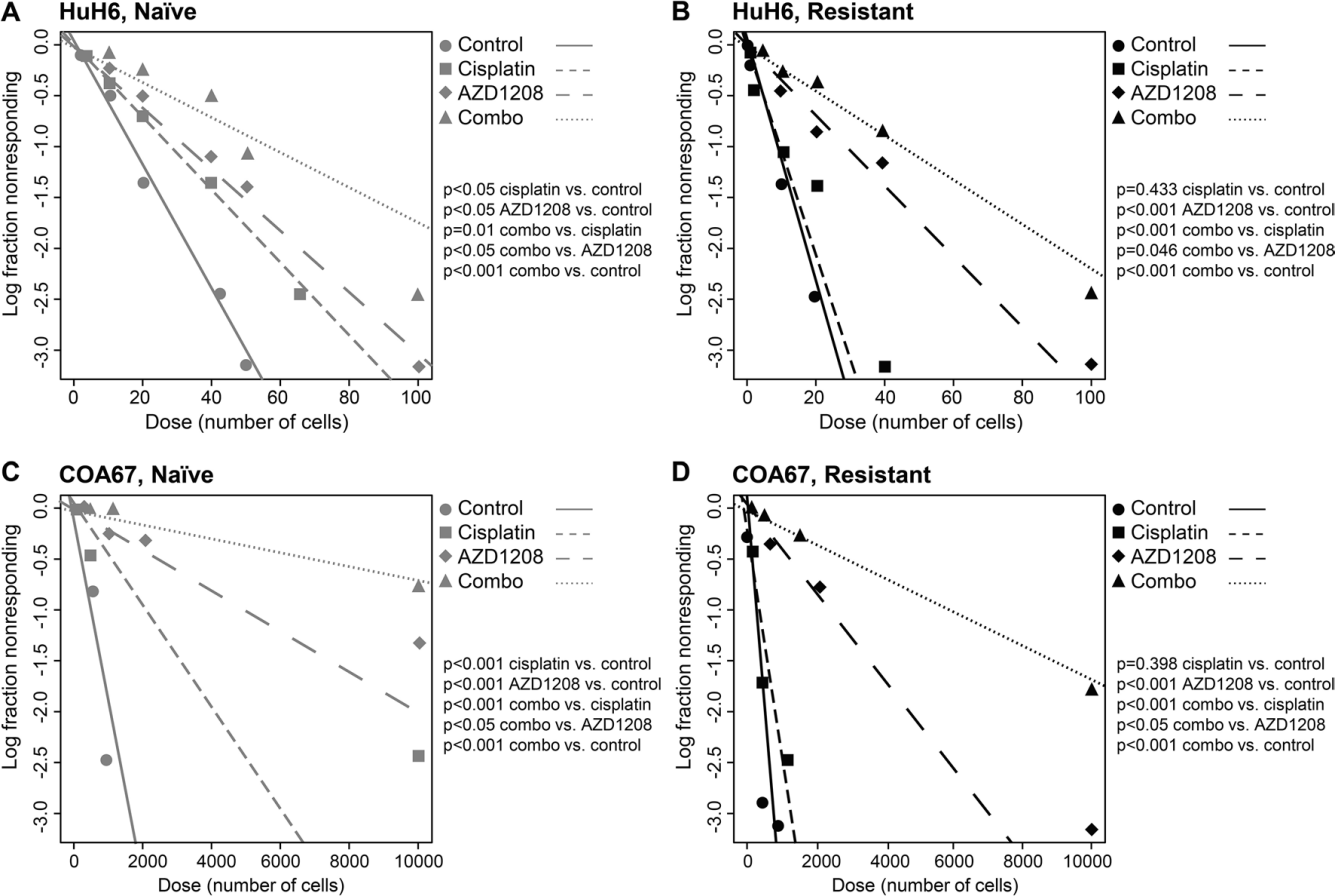
AZD1208 combined with cisplatin reduces the stem cell-like cancer cell (SCLCC) phenotype. HuH6 (**A**) cisplatin-naïve and (**B**) cisplatin-resistant and COA67 (**C**) cisplatin-naïve and (**D**) cisplatin-resistant cells were plated in non-adherent culture conditions at decreasing cell concentrations (from 5000 to 1 cell per well for HuH6 and 10,000 to 1 cell per well for COA67) and treated with 10 µM cisplatin and/or 5 µM AZD1208. After 7 days, wells were examined by a single blinded researcher and the numbers of wells containing spheres were counted. Sphere forming ability was calculated utilizing the extreme limiting dilution software (http://www.bioinf.wehi.edu.au/software/elda/) and a plot of the log proportion of negative cultures vs. the number of cells plated is shown. The slope of the line is the estimated log-active SCLCC fraction. In both hepatoblastoma lines, cisplatin treatment did not affect sphere formation in resistant cells (short dashed lines, **B**,**D**), but decreased sphere formation in cisplatin-naïve cells (short dashed lines, **A**,**C**, p < 0.05). Treatment with AZD1208 led to decreased sphere formation in both cisplatin-naïve and cisplatin-resistant HuH6 and COA67 cells (*long dashed lines*, p < 0.05). Combination of AZD1208 and cisplatin significantly reduced sphere formation in both naïve and resistant HuH6 (dotted lines, **A**,**B**) and COA67 (dotted lines, **C**,**D**) cells compared to cisplatin or AZD1208 alone, or to untreated controls (p < 0.05).

To evaluate whether PIM inhibition is able to reduce the enriched SCLCC phenotype seen in the cisplatin-resistant cells, we evaluated tumorsphere formation as well as expression of common stem cell markers following treatment with AZD1208 and/or cisplatin. Tumorsphere formation was assessed after treatment with both cisplatin (10 µM) and AZD1208 (5 µM). Cisplatin treatment decreased sphere formation in cisplatin-naïve HuH6 and COA67 cells (p < 0.05, Fig. 5A,C) but did not affect sphere formation in cisplatin-resistant HuH6 or COA67 cells (Fig. 5B,D). Treatment with AZD1208 led to decreased sphere formation in both cisplatin-naïve and cisplatin-resistant HuH6 and COA67 cells (p < 0.05, Fig. 5A–D). Treatment with both AZD1208 and cisplatin treatment resulted in decreased sphere forming ability of both HuH6 and COA67 cisplatin-naïve and cisplatin-resistant cells compared to either AZD1208 or cisplatin alone, or to untreated controls (p < 0.05, Fig. 5A–D), indicating a reduction in the SCLCC phenotype. Estimated and 95% confidence intervals for the 1/(stem cell frequency) for each group corresponding to the ELDA plots are shown in Supplementary Info Figure S1D. Notably, there was no difference in sphere forming ability of naïve and resistant cells treated with both drugs, further demonstrating the ability of AZD1208 to reduce sphere formation in cisplatin-resistant cells (Supplementary Info Figure S4).

**Figure 6:**
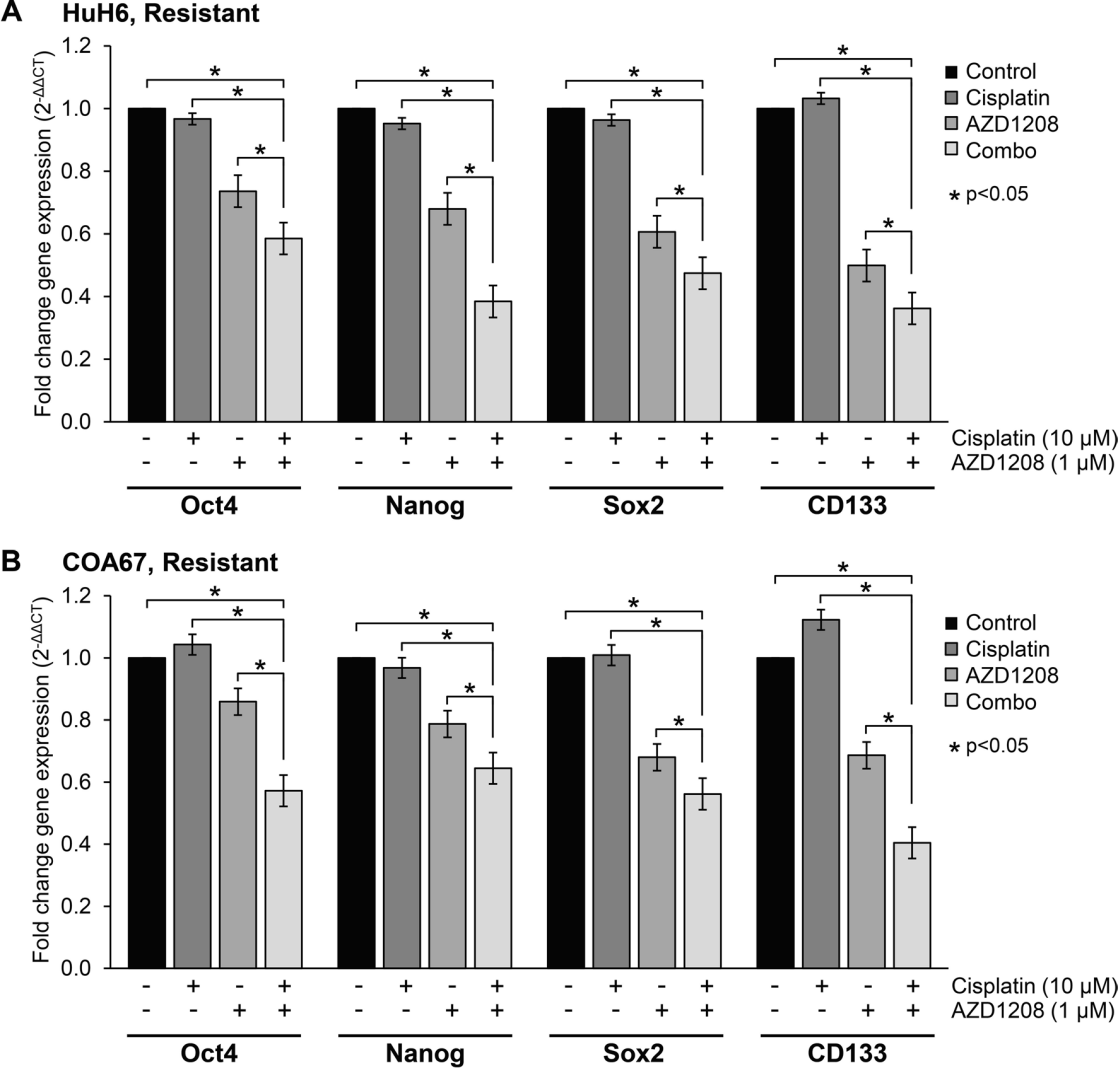
AZD1208 combined with cisplatin downregulates gene expression of known stem cell-like cancer cell (SCLCC) markers in cisplatin-resistant hepatoblastoma cells. (**A**) HuH6 and (**B**) COA67 cisplatin-resistant cells were treated with 1 µM of AZD1208, 10 µM of cisplatin, or both drugs for 72 h, and quantitative real-time PCR was utilized to assess the mRNA abundance of Oct4, Nanog, Sox2, and CD133. Relative abundance of mRNA was calculated using the ΔΔCt method and reported as mean ± SEM of three biologic replicate experiments. Cisplatin treatment did not affect mRNA abundance of the stem cell markers Oct4, Nanog, and Sox2 and upregulated the gene expression of CD133. Treatment with both AZD1208 and cisplatin significantly decreased mRNA abundance of the stem cell markers Oct4, Nanog, Sox2, and CD133 in both (**A**) HuH6 and (**B**) COA67 cisplatin-resistant cells compared to cisplatin or AZD1208 alone, or to untreated controls (p < 0.05).

To further corroborate the ELDA findings in cisplatin-resistant cells, we used qPCR to examine the mRNA abundance of the three stemness markers, Oct4, Nanog, and Sox2 in HuH6 and COA67 cisplatin-resistant cells following treatment with cisplatin (at 10 µM) and/or AZD1208 (at 1 µM). Cisplatin treatment did not significantly alter the mRNA abundance of the three transcription factors, but the addition of AZD1208 to cisplatin treatment resulted in decreased mRNA abundance of all three stemness markers in both HuH6 and COA67 cisplatin-resistant cells compared to either AZD1208 or cisplatin alone, or to untreated controls (p < 0.05, Fig. 6A,B). CD133 gene expression was also evaluated using qPCR and found to be significantly downregulated in both HuH6 and COA67 cisplatin-resistant cells treated with both AZD1208 and cisplatin, as demonstrated by the decreased mRNA abundance compared to either drug alone, or to untreated controls (p < 0.05, Fig. 6A,B). Expression of CD133 was also evaluated at the protein level by immunoblotting in COA67 cisplatin-resistant cells. Treatment with both AZD1208 and cisplatin significantly decreased CD133 protein expression in COA67 cisplatin-resistant cells compared to either cisplatin or AZD1208 alone, or to untreated controls (Supplementary Info Figure S5). These findings support those of the ELDA and indicate that the addition of AZD1208 reduces the SCLCC phenotype seen with cisplatin resistance.

### Overexpression of PIM3 in hepatoblastoma cells results in increased stem cell-like cancer cell (SCLCC) phenotype and decreased sensitivity to cisplatin

**Figure 7:**
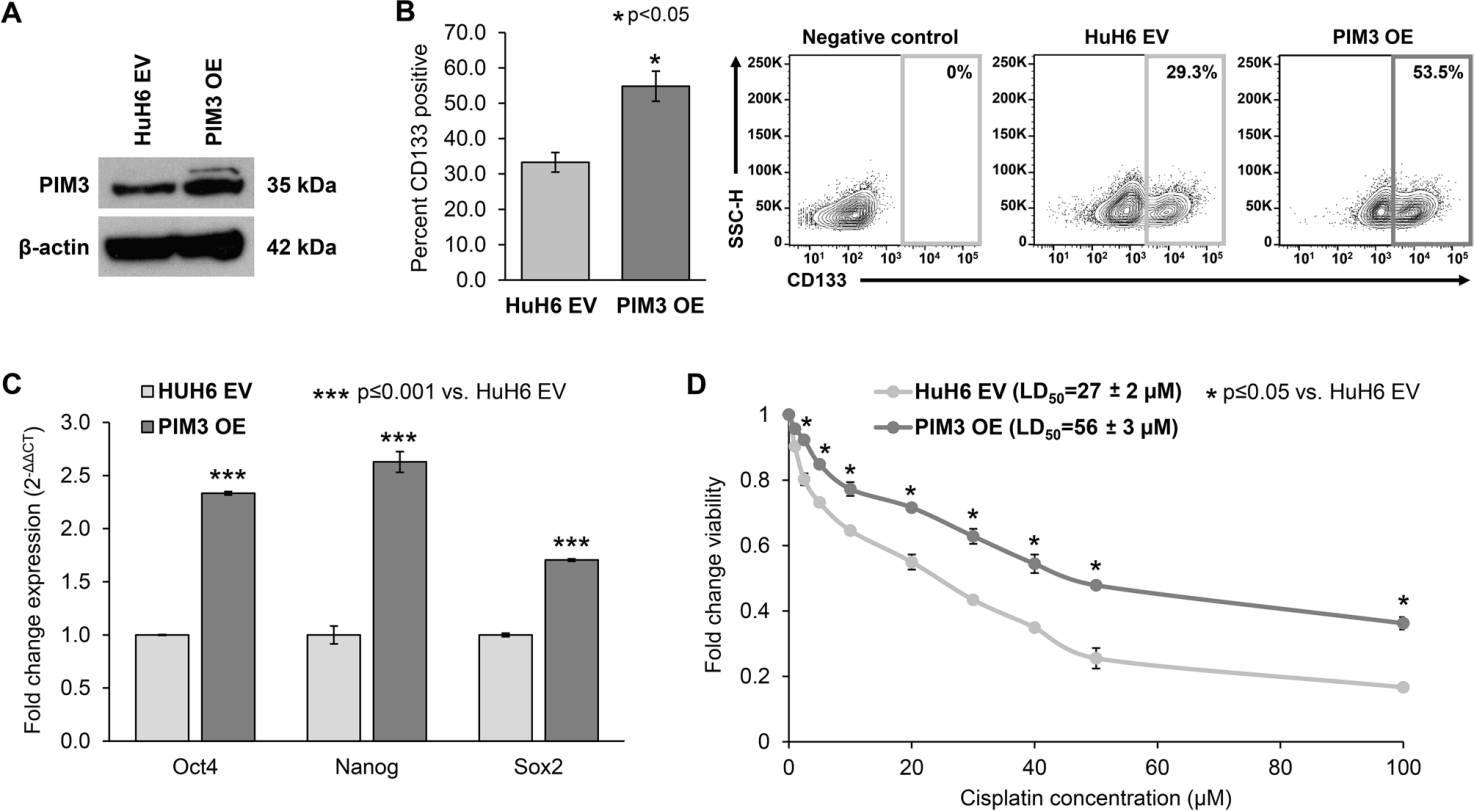
Overexpression of PIM3 in hepatoblastoma cells results in increased stem cell-like cancer cell (SCLCC) phenotype and decreased cisplatin sensitivity. HuH6 cells were stably transfected with a pcDNA3.1/V5-HisC-based expression vector for PIM3 overexpression (PIM3 OE) or an empty vector (EV) control. (**A**) Immunoblotting of whole cell lysates verified PIM3 overexpression. β-actin was used to confirm equal protein loading. (**B**) CD133 cell surface expression was evaluated by FACS in HuH6 EV and PIM3 OE cells and reported as percent CD133 expression ± SEM. CD133 expression was increased significantly in the PIM3 OE cells. Representative contour plots are shown to the right of the graph. (**C**) Quantitative real-time PCR was utilized to assess the mRNA abundance of Oct4, Nanog, and Sox2 in HuH6 EV and PIM3 OE cells. Relative abundance of mRNA was calculated using the ΔΔCt method and reported as mean ± SEM of three biologic replicate experiments. PIM3 OE cells had significantly increased abundance of all three stem cell markers compared to HuH6 EV cells (p < 0.001), further indicating an increase in the SCLCC phenotype following overexpression of PIM3. (**D**) The effect of PIM3 OE on hepatoblastoma cell viability following treatment with cisplatin was evaluated with alamarBlue assay. HUH6 EV and PIM3 OE cells were treated with increasing concentrations of cisplatin (0–100 µM) for 72 h and viability measured. PIM3 OE cells were significantly less sensitive to cisplatin than HuH6 EV cells, indicating that PIM3 contributes to cisplatin insensitivity.

To further support the role of PIM inhibition with AZD1208 in reducing the SCLCC phenotype and sensitizing hepatoblastoma cells to cisplatin, we sought to assess the role of the overexpression of PIM3 kinase on cancer cell stemness and sensitivity to cisplatin. PIM3 overexpression (PIM3 OE) was achieved using stable transfection of HuH6 cells with a pcDNA3.1/V5-HisC-based expression vector and confirmed by immunoblotting (Fig. 7A).

We first evaluated the cell surface expression of CD133 using flow cytometry and found that HuH6 cells overexpressing PIM3 had higher CD133 cell surface expression compared to their empty vector (EV) transfected counterparts (55 ± 4% vs. 33 ± 3%, p ≤ 0.05, Fig. 7B). Further, we evaluated the mRNA abundance of the three transcription factors, Oct4, Nanog, and Sox2, using qPCR and found that PIM3 OE cells had significantly higher mRNA abundance of all three stemness markers compared to EV cells (p ≤ 0.001, Fig. 7C).

To evaluate the effect of PIM3 on cisplatin sensitivity, we assessed cell viability following treatment with increased doses of cisplatin (0–100 µM). PIM3 OE cells were less sensitive to cisplatin compared to EV cells (LD_50_, 56 ± 3 µM vs. 27 ± 2 µM, p ≤ 0.05, Fig. 7D). Together, these findings highlight the role of PIM3 in contributing to cancer cell stemness and cisplatin insensitivity.

## Discussion

A novel aspect of this study is the development of cisplatin-resistant HuH6 and COA67 hepatoblastoma xenograft models. To our knowledge, only two other groups have established cisplatin-resistant hepatoblastoma cells in vitro and this current study is the first report describing the development of cisplatin-resistant cells using a hepatoblastoma patient-derived xenograft. Eicher et al. attempted to derive cisplatin-resistant cells using both HuH6 and HepT1 cells with escalation of cisplatin concentration in 2D culture as well as serial passage of xenografts in mice treated with cisplatin but was not successful. However, they were able to develop cisplatin-resistant cells in 3D culture conditions^24^. We had similar unsuccessful results when attempting to escalate cisplatin concentration in 2D culture of HuH6 cells. Recently, Fujiyoshi et al. described the generation of cisplatin-resistant HuH6 in 2D culture^25^. Eicher et al. compared the HuH6 cells passaged in cisplatin treated mice to the continuous HuH6 cell line grown in 2D culture, which did not account for the contribution of the tumor microenvironment. In contrast, we utilized serial xenograft tumors passed in non-treated mice as controls, which may have contributed to the success of our resistance xenograft model. Additionally, Eicher et al. performed experiments after the xenograft tumor cells had been passed in culture, again potentially altering the environment of the cells and affecting their ability to maintain resistance to cisplatin. We utilized the cells immediately after explantation and dissociation, as we discovered that the cells did lose their cisplatin resistance after subsequent exposure to standard tissue culture environment. The degree of cisplatin resistance in the HuH6 cells in our study was similar to that achieved by the other two investigators^24,25^. The use of a hepatoblastoma PDX model to establish cisplatin resistance is an added strength of the current study since PDXs may more closely recapitulate the clinical condition than cancer cell lines in culture^20^.

We demonstrated that PIM3 expression was increased in cisplatin-resistant versus cisplatin-naïve cells, indicating that PIM3 plays a role in chemoresistance. Other investigators have reported that PIM family kinases reduce the efficacy of chemotherapy in cancer. PIM inhibition with the pan-PIM inhibitor, SGI-1776, sensitized pancreatic ductal adenocarcinoma cells to gemcitabine and led to an increased gemcitabine-induced apoptosis^26^. SGI-1776 also sensitized prostate cancer cells to taxane-based therapies^27^. Chen et al. found that overexpression of PIM1 rendered pancreatic cells resistant to cisplatin underscoring the role of PIM family kinases in mediating chemoresistance^28^. PIM3, specifically, has been shown to play a role in mediating chemoresistance. Guo et al. reported that PIM3 was one of three proteins whose expression was most increased in adriamycin- and vincristine-resistant gastric cancer cell lines and silencing of PIM3 reversed the adriamycin-resistant phenotype^29^. In hepatocellular carcinoma, PIM3 expression was increased in the tumors of those who received chemotherapy and PIM3 induced multi-drug resistance in vitro^30^.

Since PIM3 expression was increased in the cisplatin-resistant cells, we sought to determine whether combining the pan-PIM inhibitor, AZD1208, to cisplatin would more effectively decrease proliferation of cisplatin-resistant hepatoblastoma cells. We chose AZD1208 for the current study as it is a highly selective ATP-competitive PIM kinase inhibitor^31^, is orally bioavailable, and has been evaluated in Phase I clinical trials in adults with advanced solid malignancies^32^, potentially leading to earlier translation to clinical application. The addition of 1 µM of AZD1208 to cisplatin in HuH6 and COA67 cisplatin-resistant cells decreased their proliferation to levels of cisplatin-naïve cells treated with cisplatin alone, indicating that by targeting PIM3, previously resistant cells could be sensitized to cisplatin. While other researchers have found that PIM inhibition sensitized other cancer cells to chemotherapy^27,29^, the current study is the first to report sensitization in hepatoblastoma cells. This finding is important with clinical relevance since cisplatin is the first line of treatment for hepatoblastoma, and tumor resistance to this drug continues to pose a challenge and contribute to poor outcomes.

To determine a potential mechanism by which PIM inhibition enhanced the ability of cisplatin to kill resistant cells, we examined the ability of AZD1208 to induce apoptosis in those cells. Kinome profiling revealed that phosphorylation of BAD was increased in cisplatin-resistant hepatoblastoma tumors compared to their naïve counterparts. These findings suggested that phosphorylation of BAD, which inhibits its pro-apoptotic activity, may be responsible for the cell death resistance seen in cisplatin-resistant hepatoblastoma model. PIM kinases have been shown to phosphorylate BAD at Ser 112 to antagonize drug-induced apoptosis^28^. We previously demonstrated that PIM3 phosphorylated BAD in hepatoblastoma impeding apoptosis and promoting tumorigenesis^20^. Since we found both increased PIM3 expression and activation of a known PIM3 target in cisplatin-resistant cells, we hypothesized that PIM3 inhibition would potentiate cisplatin-induced apoptosis in these cells. We showed that treatment with a PIM kinase inhibitor resulted in an increase of both early and late cisplatin-induced apoptosis in resistant hepatoblastoma cells, overcoming drug-induced resistance to cell death and restoring the effectiveness of cisplatin.

The data presented in this study postulate a mechanism by which cisplatin chemoresistance might occur—through an increase in the SCLCC phenotype. We have previously characterized the hepatoblastoma SCLCC subpopulation and identified CD133 as a cell surface marker for SCLCC in hepatoblastoma^15^. Here we report that cisplatin resistance increased the percentage of hepatoblastoma cells expressing CD133 and increased their sphere-forming ability when compared to cisplatin-naïve cells. These results imply an increased percentage of SCLCCs in cisplatin-resistant tumors, likely due to selective targeting of cisplatin sensitive non-SCLCCs, such that more of the chemoresistant SCLCCs remain. These findings are consistent with those reported by other investigators. In hepatocellular carcinoma, low-dose cisplatin decreased cell survival, but the surviving cells expressed CD133 at a higher rate^19^. Similar findings were seen in ovarian cancer where cisplatin was found to enrich SCLCCs^18^. Treatment of lung cancer cells with cisplatin both in vitro and in vivo increased CD133 expression. Furthermore, CD133 expression was higher in relapsed tumors in lung cancer patients who had received cisplatin therapy^17^. We also described a strategy to potentially overcome platinum-based chemoresistance in hepatoblastoma by targeting the SCLCC population. We found that the addition of the PIM inhibitor AZD1208 to cisplatin-resistant cells abrogated the increase in SCLCCs as noted by the decreased tumorsphere formation, the decreased mRNA abundance of the stem cell markers Oct4, Nanog, Sox2, and the decreased CD133 expression. These findings highlight the premise of PIM inhibition as an adjunct for the treatment of cisplatin-insensitive hepatoblastoma, by targeting the chemoresistant SCLCC population and significantly reducing the SCLCC frequency which, along with the decreased proliferation and increased cisplatin-induced apoptosis of the non-SCLCC population, could ideally lead to an improved or renewed response to cisplatin.

We can speculate the role of PIM3 in mediating cisplatin resistance in hepatoblastoma. Perhaps the increase in PIM3 is similar to that of SCLCC phenotype, in that treatment with cisplatin selects for the cell population with greater PIM3 expression, which in turn induces stemness and resistance to apoptosis. This concept is consistent with our current and previous findings that PIM3 overexpression maintains the SCLCC subpopulation and drives a more aggressive phenotype associated with worse patient prognosis^33^. We showed that PIM3 overexpression increased cell surface expression of CD133 and mRNA abundance of stemness markers, and was associated with cisplatin insensitivity in hepatoblastoma cells. In pancreatic cancer, PIM3-induced overexpression of stemness markers, including Oct4, was associated with resistance to gemcitabine^34^. Other authors have put forth the hypothesis that PIM kinases promote drug resistance through induction of multi-drug resistance mechanisms. Guo et al. indirectly showed that targeting PIM3 in hepatocellular carcinoma cells decreased expression of the multi-drug resistance proteins Pgp, MRP3, and MRP2^30^. It has also been reported that PIM1 expression was correlated with the expression of ATP-binding cassette (ABC) drug efflux proteins and that SGI-1776 directly inhibited ABC proteins to sensitize cells to chemotherapy^35^. PIM2 and PIM3 did not appear to be correlated with ABC protein expression, although these studies focused on leukemia and myeloma, in which PIM1 is known to play a larger role^36,37^.

In conclusion, PIM inhibition with AZD1208 sensitized cisplatin-resistant cells to cisplatin, enhanced cisplatin-mediated apoptosis, and decreased the SCLCC phenotype by decreasing the SCLCC frequency found to be enriched with cisplatin resistance. These findings suggest that PIM inhibition overcomes hepatoblastoma tumor chemoresistance which is exciting given that resistance to standard chemotherapeutics remains a significant barrier to effective treatment. PIM inhibition, in combination with the standard chemotherapy agent cisplatin, could potentially be employed in the clinical setting for treatment of refractory or relapsed disease.

## Methods

### Cells and cell culture

The human long-term passaged hepatoblastoma cell line, HuH6, was acquired from Thomas Pietschmann (Hannover, Germany)^38^ and maintained in Dulbecco’s Modified Eagle’s Medium supplemented with 10% fetal bovine serum (HyClone, GE Healthcare Life Sciences, Logan, UT), 1 µg/mL penicillin/streptomycin (Gibco, Carlsbad, CA), and 2 mmol/L l-glutamine (Thermo Fisher Scientific, Waltham, MA). The human hepatoblastoma patient-derived xenograft (PDX), COA67, was developed as previously described^20^. Briefly, under ethical approval from the University of Alabama at Birmingham Institutional Review Board (X130627006) and Institutional Animal Care and Use Committee (IACUC-09803) and in accordance with the Declaration of Helsinki and the guidelines of the National Institutes of Health, and following written informed consent, human hepatoblastoma tumor tissue was obtained from a patient with primary hepatoblastoma undergoing surgical excision. Fresh tissue was kept in serum-free Roswell Park Memorial Institute (RPMI) 1640 medium on ice for transport and subsequently transplanted in a sterile fashion subcutaneously in the flank of female NOD SCID mice (Envigo, Prattville, AL) under anesthesia with 3% inhalational isoflurane^20^. Tumor volumes were measured and when tumors reached IACUC parameters, mice were euthanized and tumors were harvested and a portion sequentially passed into another mouse to maintain the PDX line. Individual cells were obtained by dissociating the COA67 PDX tumors using the Papain Dissociation System (Worthington Biochemical Corporation, Lakewood, NJ). COA67 cells were maintained in Dulbecco’s Modified Eagle’s Medium/Ham’s F12 supplemented with 1 µg/mL penicillin/streptomycin (Gibco), 2 mmol/L l-glutamine (Thermo Fisher Scientific), 20 ng/mL epidermal growth factor (EMD Millipore, Billerica, MA), 20 ng/mL β-fibroblast growth factor (EMD Millipore), 2% B27 supplement (Gibco), and 2.5 µg/mL amphotericin B (HyClone). Both HuH6 and COA67 cell lines were verified within the last 12 months using short tandem repeat analysis (Heflin Center for Genomic Sciences, UAB, Birmingham, AL). Real-time qPCR was performed to assess the percentage of human and mouse DNA contained in the COA67 PDX to ensure that the tumor did not contain mouse contamination (TRENDD RNA/DNA Isolation and TaqMan QPCR/Genotyping Core Facility, UAB, Birmingham, AL). All cells were maintained in culture at 37 °C and 5% CO_2_ and determined to be free of Mycoplasma.

### Antibodies and reagents

Rabbit monoclonal anti-PIM3 (4165) and polyclonal anti-vinculin (4650) was obtained from Cell Signaling Technology (Beverly, MA). Mouse monoclonal anti-CD133 (ab19898) was obtained from Abcam (Cambridge, MA). Mouse anti-GAPDH (MAB374, clone 6C5) was obtained from Millipore (EMD Millipore, Billerica, MA). Mouse monoclonal anti-β-actin (A1978) was from Sigma Aldrich (St. Louis, MO). AZD1208 was purchased from Selleck Chemicals (Houston, TX) and cisplatin from Cayman Chemical (Cayman Chemical, Ann Arbor, MI).

### Development of cisplatin-resistant human hepatoblastoma cells

Experiments were approved by the University of Alabama at Birmingham Institutional Animal Care and Use Committee (IACUC-09803) and conducted within institutional, national, and international guidelines and in compliance with the Animal Research: Reporting of In Vivo Experiments (ARRIVE) guidelines. Six-week old female athymic nude mice (Charles River, Frederick, MD) were maintained in the specific pathogen-free facility with standard 12-h light/dark cycles and access to chow and water ad libitum. Cisplatin-resistant hepatoblastoma cells were developed as depicted in Fig. 1 A. Briefly, HuH6 cells were injected subcutaneously into the flank of one mouse and tumor volume was measured twice weekly using calipers and calculated with the standard formula (width^2^ x length)/2, where the length is the largest measurement. Once tumor volume reached approximately 100 mm^3^, the mouse was treated with 2 mg/kg/day cisplatin via intraperitoneal injection for three consecutive days weekly.

The COA67 PDX cisplatin-resistant model was developed in the same fashion except 2 mm^3^ chunks of tumor were implanted into the animal’s flank instead of a single cell suspension. Once tumor volume reached approximately 300 mm^3^, the animal was treated with 1 mg/kg/day cisplatin via intraperitoneal injection for two consecutive days weekly.

Animals were humanely euthanized with CO_2_ and cervical dislocation when the tumor reached approximately 1500 mm^3^. All tumors were harvested and portions were (i) frozen for lysates and RNA, (ii) dissociated for in vitro studies to yield cells that were referred to as “cisplatin-resistant” cells, and (iii) chopped for re-implantation into a second animal. Frozen tumor pieces were stored at − 80 °C. Tumors were dissociated to a single cell solution using a human Tumor Dissociation Kit (Miltenyi, Bergisch Gladbach, Germany). Chopped pieces were placed in 25% Matrigel (25%, Corning, Corning, NY) prior to passage. When this second generation tumor grew and reached an appropriate volume (100 mm^3^ for HuH6 and 300 mm^3^ for COA67), treatment with cisplatin was initiated as above. After at least six serial passages, cells were sufficiently insensitive to cisplatin to be deemed “cisplatin-resistant” cells.

Alternatively, tumors from mice bearing HuH6 or COA67 xenografts that followed the same cycle as described above, but were not treated with cisplatin, yielded HuH6 and COA67 cells that were referred to as “cisplatin-naïve” and served as controls.

### Viability and proliferation assays

The alamarBlue Cell Viability Assay (Thermo Fisher Scientific) was used to measure cell viability. HuH6 cisplatin-resistant or cisplatin-naïve cells (1.5 × 10^3^ cells per well) were plated in 96-well plates, allowed to attach overnight, and treated with cisplatin at increasing concentrations (0 to 30 µM). COA67 cisplatin-resistant or cisplatin-naïve cells (1.5 × 10^4^ cells per well) were plated in 96-well plates, allowed to rest for 4 h, and treated with increasing concentrations of cisplatin (0 to 30 µM). Following 72 h of treatment, 10 µL of alamarBlue reagent was added to each well and the absorbance was measured at excitation wavelength of 562 nm and emission wavelength of 595 nm using a microplate reader (BioTek Gen5, BioTek, Winooski, VT). Viability was reported as mean fold change ± standard error of the mean (SEM).

The CellTiter 96 Aqueous Non-Radioactive Cell Proliferation Assay (Promega, Madison, WI) was used to measure proliferation. HuH6 cisplatin-resistant or cisplatin-naïve cells (5 × 10^3^ per well) were plated in 96-well plates, allowed to attach overnight, and treated with cisplatin at increasing concentrations (0 to 30 µM). COA67 cisplatin-resistant or cisplatin-naïve cells (3 × 10^4^ cells per well) were plated in 96-well plates, allowed to rest for 4 h, and treated with increasing concentrations of cisplatin (0 to 30 µM). After 72 h of treatment, 10 µL of CellTiter 96 reagent was added to each well and the absorbance was measured at 490 nm using a microplate reader (BioTek Gen5). Proliferation was reported as mean fold change ± SEM.

### Flow cytometry

Cisplatin-resistant and cisplatin-naïve cells were labeled with anti-CD133/1(AC133)-APC (Miltenyi) according to the manufacturer’s instructions. The percent of APC positive cells was determined via flow cytometry using the BD FACSCalibur platform (BD Biosciences, Franklin Lakes, NJ) and the Attune NxT Flow Cytometer (Invitrogen, Thermo Fisher, Eugene, OR). Unlabeled cells were used as negative controls. Apoptosis was evaluated using the FITC Annexin V Apoptosis Detection Kit (BD Biosciences, San Jose, CA) according to manufacturer’s protocol. Cells were treated with either cisplatin alone (10 µM), AZD1208 alone (1 µM), or both cisplatin and AZD1208 for 72 h (for HuH6) and 24 h (for COA67). Cells were collected, washed with cold PBS, centrifuged at 1200 rpm for 4 min, and re-suspended in 1 × binding buffer (0.1 M HEPES/NaOH, 1.4 M NaCl, 25 mM CaCl2, pH 7.4). Annexin V-FITC and propidium iodide (PI) staining solutions were added and cells were incubated for 15 min at room temperature (20–25 °C) in the dark. Additionally, cells were unstained, stained with PI only, or stained with Annexin V only and used for negative control and compensation. The percent of FITC and/or PI positive cells was determined via flow cytometry using the Attune NxT Flow Cytometer (Invitrogen). Data are represented as percent cell population with cells not stained with Annexin V or PI representing live cells, Annexin V + PI− stained cells representing early apoptotic cells, Annexin V + PI + cells representing late apoptotic cells, and cells stained with PI only representing necrotic or dead cells. All flow cytometry analyses were performed using FlowJo software (BD Biosciences).

### In vitro extreme limiting dilution analysis (ELDA)

Cells were plated into 96 well ultra-low attachment plates using serial dilutions from 5000 to 1 cell per well for cisplatin-resistant and cisplatin-naïve HuH6 cells with at least 12 replicates per dilution. Serial dilutions of 10,000 to 1 cell per well were used for cisplatin-resistant and cisplatin-naïve COA67 cells. Cells were plated into Dulbecco’s Modified Eagle’s Medium/Ham’s F12 supplemented with 2 mmol/L l-glutamine (Thermo Fisher Scientific), 1 µg/mL penicillin/streptomycin (Gibco), 20 ng/mL epidermal growth factor (EMD Millipore), 20 ng/mL beta-fibroblast growth factor (EMD Millipore), 2% B27 supplement (Gibco), and 2.5 µg/mL amphotericin B (HyClone) in the presence or absence of 10 µM cisplatin, 5 µM AZD1208, or both. Once spheres were present in the wells containing the most cells, all wells were counted. The presence or absence of spheres in each well was quantified by a single blinded researcher. Extreme limiting dilution analysis software was utilized to analyze the data (http://bioinf.wehi.edu.au/software/elda/)^39^ and a plot of the log proportion of negative cultures vs. the number of cells plated is generated. The slope of the line is the estimated log-active SCLCC fraction. Tables showing estimated and 95% confidence intervals for the 1/(stem cell frequency) for each group are also generated.

### Quantitative real-time PCR (qPCR)

Total cellular RNA was extracted using the RNeasy kit (Qiagen, Germantown, MD) according to the manufacturer's protocol. For synthesis of cDNA, 1 µg of RNA was used in a 20 µl reaction mixture utilizing an iScript cDNA Synthesis kit (Bio-Rad, Hercules, CA) according to the supplier's instructions. Resulting reverse transcription products were stored at − 20 °C until further use. For quantitative real-time PCR, SsoAdvanced SYBR Green Supermix (Bio-Rad) was utilized according to manufacturer's protocol. Probes specific for the transcription factors Oct4, Nanog, Sox2, CD133, and NOTCH1 as well as for β-actin were obtained (Applied Biosystems, Foster City, CA). Primers for ALDH1A3, NOTCH2, NOTCH3, and Hes1 were designed using Primer3 (web version 4.1.0)^40^ and the basic local alignment search tool (BLAST, NCBI) was used to check for non-specific binding. Sequences for these primers are included in the Supplementary Info (Figure S2 B). qPCR was performed with 10 ng cDNA in 50 µL reaction volume. Amplification was done using an Applied Biosystems 7900HT cycler (Applied Biosystems) and cycling conditions were 95 °C for 2 min, followed by 39-cycle amplification at 95 °C for 5 s and 60 °C for 30 s. Samples were analyzed in triplicate with β-actin utilized as an internal control. Relative gene expression was calculated using the ΔΔCt method^41^ and reported as mean fold change ± SEM.

### Kinome assay

Paired cisplatin-resistant and cisplatin-naïve tumors from HuH6 and COA67 xenografts were utilized. Kinomic profiling of 2–15 µg of protein lysates (lysed in M-Per lysis buffer, Pierce Scientific, Waltham, MA) containing 1:100 Halt’s protease and phosphatase inhibitors (Pierce cats. 78420, 78415) was conducted on the PamStation 12 platform, manufactured by PamGene (‘s-Hertogenbosch, Netherlands) and analyzed within the UAB Kinome Core (https://www.uab.edu/medicine/radonc/research/labs-core-facilities/kinome-core) as described previously^42^. After protein quantification (BCA protein determination, Pierce Scientific), total soluble protein lysates were loaded onto the appropriate PamChip [PTK (tyrosine kinome, article code 86312) or STK (serine/threonine kinome, article code 87102)] in kinase buffer^43,44^. This platform utilizes a high throughput peptide microarray system analyzing 144 individual tyrosine phosphorylatable peptides, or 144 serine and threonine phosphorylatable peptides imprinted and immobilized in a 3D format to assess kinomic activity in cell or tissue lysates. As such, molecular profiles of samples were measured by kinase activity against 288 phosphorylatable peptides (phosphopeptides) containing a distinct 12–15 amino acid sequence (comprising over 560 phosphorylatable tyrosine, serine, or threonine residues in total). FITC conjugated phosphospecific antibodies (PamGene) were used for visualization during and after lysates were pumped through the array. Capture of peptide phosphorylation signal was via a computer-controlled CCD camera. Kinomic profiling was analyzed using software including Evolve2 (PamGene) for array processing and image capture, and BioNavigator v6.0 (PamGene) for raw data transformation into kinetic (initial velocity) and steady state (postwash) values. Peptide spot intensity (brightness) was captured across multiple exposure times (10–200 ms) and the slope was taken, multiplied by 100, log2 transformed, and used as signal. Raw data were included in the Supplementary Info Table S1. Unsupervised hierarchical clustering of peptide signals for each sample was performed in BioNavigator and displayed as a heatmap. The peptide lists within the clusters were then analyzed for upstream kinase prediction (UpKin PamApp; PTK v6.0, STK v6.0) using scoring from Kinexus Phosphonet (phosphonet.ca) to generate kinase statistics and specificities as previously described^43,44^.

### Immunoblotting

Cells or homogenized xenograft specimens were lysed on ice for one hour in radio-immunoprecipitation assay (RIPA) buffer supplemented with protease inhibitors (Sigma Aldrich), phosphatase inhibitors (Sigma Aldrich), and phenyl-methane-sulfonyl-fluoride (Sigma Aldrich). Lysates were centrifuged at 14,000 rpm for 1 h at 4 °C. The Pierce BCA Protein Assay reagent (Thermo Fisher Scientific) was used to determine protein concentrations. Lysates were then separated by electrophoresis on sodium dodecyl sulfate polyacrylamide (SDS-PAGE) gels. Precision Plus Protein Kaleidoscope Standards (Bio-Rad) were used to confirm the expected size of the target proteins. Antibodies were used according to the manufacturers’ recommended conditions. Luminata Classico or Crescendo Western HRP Substrate (EMD Millipore) were used to develop the immunoblots. Blots were stripped with stripping buffer (Bio-Rad) at 65 °C for 20 min and then re-probed with antibodies to proteins of interest. GAPDH, β-actin, or vinculin were used to confirm equal protein loading.

### Stable overexpression of PIM3

The PIM3 expression vector, pcDNA3.1/V5-His-*PIM3*, was a generous gift from Dr. Jussi Taipale and was generated by PCR amplification and cloning into the pcDNA3.1/V5-HisC vector^45^. The plasmid was sequenced for verification (Genomics Core, UAB). The resulting construct (pcDNA3.1/V5-His-*PIM3) w*as transfected into HuH6 cells to induce PIM3 overexpression (PIM3 OE) using FuGENE HD Transfection Reagent (Promega) per the manufacturer’s protocol. Empty vector (EV, pcDNA3.1/V5-HisC) was used as a control. Briefly, HuH6 cells were plated on the day prior to transfection. The appropriate plasmid was incubated for 15 min at room temperature in Opti-MEM™ media (Thermo Fisher Scientific) with FuGENE HD Transfection Reagent (Promega) in a 3:2 ratio of transfection reagent to DNA with 7.5 µg DNA per 1 × 10^6^ cells and added to the cells while swirling the flask. Cells were transfected with either EV or PIM3 OE vector and selected with Geneticin™ (G-418, Sigma Aldrich, at 2 mg/ml) starting at 48 h after transfection. Lysates were made to perform immunoblotting, as described above, to assess for PIM3 overexpression.

To assess sensitivity to cisplatin, HuH6 EV or PIM3 OE cells (1.5 × 10^3^ cells per well) were plated in 96-well plates, allowed to attach overnight, and treated with cisplatin at increasing concentrations (0 to 100 µM) for 72 h. Viability was assessed using the alamarBlue Cell Viability Assay as described above.

### Statistical analysis

All experiments were performed at a minimum of three biologic replicates. Data were reported as mean ± SEM of separate experiments. Student's t-test or analysis of variance (ANOVA) was used to compare means between the groups as appropriate. Statistical significance was defined as p ≤ 0.05.

## Supplementary Information

Supplementary Information.

Supplementary Table S1.

## Data Availability

All data generated or analyzed during this study are included in this published article and its supplementary information files.
