# Supplementary material for: PIM kinases mediate resistance to cisplatin chemotherapy in hepatoblastoma
Source: Sci Rep. 2021 Mar 16;11:5984. doi: 10.1038/s41598-021-85289-0 (PMC7966748; doi:10.1038/s41598-021-85289-0)
Supplement: Supplementary file 2 — Supplementary Table S1. [file 41598_2021_85289_MOESM2_ESM.pdf]

PIM Kinases Mediate Resistance to Cisplatin Chemotherapy in Hepatoblastoma

Raoud Marayati, M.D.[1*], Laura L. Stafman, M.D., Ph.D.[1*], Adele P. Williams, M.D.[1], Laura V. Bownes M.D.[1], Colin H. Quinn, B.S.[1], Jamie M. Aye, M.D.[2], Jerry E. Stewart, B.S.[1], Karina J. Yoon, Ph.D.[3], Joshua C. Anderson, Ph.D.[4], Christopher D. Willey, M.D, Ph.D.[4], Elizabeth A. Beierle, M.D.[1†]

[1]Department of Surgery, University of Alabama at Birmingham, Birmingham, Alabama 35233, USA

[2]Department of Pediatric Hematology Oncology, University of Alabama at Birmingham, Birmingham, Alabama 35233, USA

[3]Department of Pharmacology and Toxicology, University of Alabama at Birmingham, Birmingham, Alabama 35233, USA

[4]Department of Radiation Oncology, University of Alabama at Birmingham, Birmingham, Alabama 35233, USA

*These authors contributed equally to this work.

[†]Corresponding Author.

Supplementary Information

Table_S1_BeierlePTKSTK.xlsx (uploaded separately)

Table S1. Complete kinomic data from paired cisplatin-resistant and cisplatin-naïve tumors from HuH6 and COA67 xenografts. This file (***Table_S1_BeierlePTKSTK.xlsx***) contains the raw data with median peptide phosphorylation signal minus background for each image captured, over increasing cycles, and across multiple exposure times (10-200 ms) from both the tyrosine kinase and serine/threonine kinase analyses provided.

Figure S1

A

Dose	Tested	Response	Group
**100**	24	24	HuH6 Naïve
**50**	24	24	HuH6 Naïve
**40**	24	24	HuH6 Naïve
**20**	24	21	HuH6 Naïve
**10**	24	20	HuH6 Naïve
**1**	24	5	HuH6 Naïve
**100**	24	24	HuH6 Resistant
**50**	24	24	HuH6 Resistant
**40**	24	22	HuH6 Resistant
**20**	24	19	HuH6 Resistant
**10**	24	8	HuH6 Resistant
**1**	24	1	HuH6 Resistant

Dose	Tested	Response	Group
**1000**	24	24	COA67 Naïve
**500**	24	22	COA67 Naïve
**100**	24	2	COA67 Naïve
**50**	24	0	COA67 Naïve
**40**	24	0	COA67 Naïve
**20**	24	0	COA67 Naïve
**10**	24	0	COA67 Naïve
**1**	24	0	COA67 Naïve
**1000**	24	24	COA67 Resistant
**500**	24	24	COA67 Resistant
**100**	24	24	COA67 Resistant
**50**	24	4	COA67 Resistant
**40**	24	0	COA67 Resistant
**20**	24	0	COA67 Resistant
**10**	24	0	COA67 Resistant
**1**	24	0	COA67 Resistant

B

Group	1/(stem cell frequency) [95% CI]
**HuH6 Naïve**	15.3 [20.06, 11.67]
**HuH6 Resistant**	6.83 [9.57, 4.87]

Group	1/(stem cell frequency) [95% CI]
**COA67 Naïve**	367 [498, 270]
**COA67 Resistant**	128 [175, 94]

C

Group	Chisq	DF	p value
**HuH6 Naïve vs. Resistant**	13.7	1	0.000212

Group	Chisq	DF	p value
**COA67 Naïve vs. Resistant**	19.3	1	1.14e-05

D

HuH6, Naïve

Group	1/(stem cell frequency) [95% CI]
**Control**	15.5 [20.7, 10.6]
**Cisplatin**	28.2 [40.4, 19.7]
**AZD1208**	31 [44.5, 21.7]
**Combo**	53.6 [78.5, 36.6]

HuH6, Resistant

Group	1/(stem cell frequency) [95% CI]
**Control**	7.18 [11.5, 4.49]
**Cisplatin**	9.22 [14.2, 5.97]
**AZD1208**	26.33 [37.3, 18.37]
**Combo**	44.04 [63.7, 30.43]

COA67, Naïve

Group	1/(stem cell frequency) [95% CI]
**Control**	446 [770, 258]
**Cisplatin**	2003 [4464, 899]
**AZD1208**	4851 [9272, 2538]
**Combo**	12731 [26886, 6028]

COA67, Resistant

Group	1/(stem cell frequency) [95% CI]
**Control**	128 [200, 82.5]
**Cisplatin**	170 [269, 108.2
**AZD1208**	1828 [4125, 809.7]
**Combo**	5901 [11270, 3089.5]

Figure S1. Extreme limiting dilution analysis (ELDA) for the assessment of sphere forming ability and the frequency of stem cell-like cancer cells (SCLCCs). (**A**) Limiting dilution data entered into the extreme limited dilution analysis (ELDA) tool corresponding to Figure 2 C. The "Dose" represents the number of cells plated in each culture, "Tested" represents the number of cultures/wells, and "Response" represents the number of cultures/wells with tumorspheres present. A plot of the log proportion of negative cultures vs. the number of cells plated is shown in Figure 2 C, with the slope of the line representing the estimated log-active SCLCC fraction. (**B**) Estimated and 95% confidence intervals (CI, with lower and upper bounds shown in brackets) for the 1/(stem cell frequency) for each group. (**C**) Tables showing the test for differences in SCLCC frequencies between the two groups. (**D**) Estimated and 95% confidence intervals (CI, with lower and upper bounds shown in brackets) for the 1/(stem cell frequency) for each group corresponding to the ELDA plots shown in Figure 5.

Figure S2

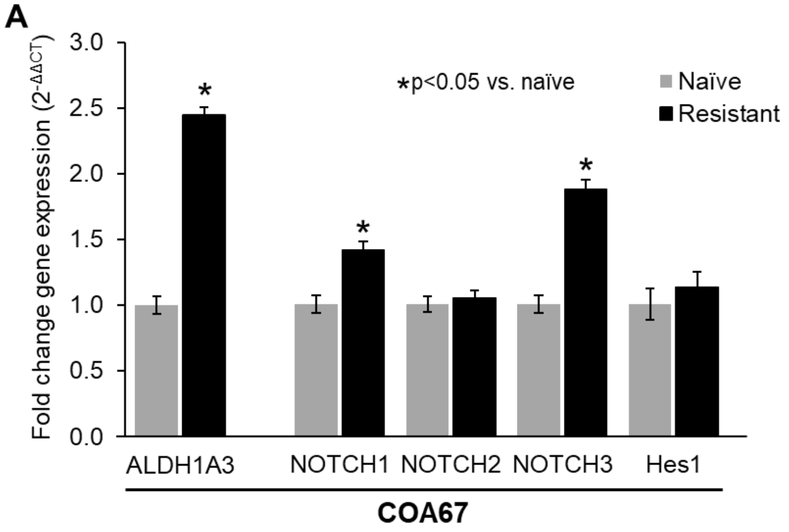

B

Gene	Forward primer (5'-3')	Reverse primer (5'-3')
ALDH1A3	TGGATCAACTGCTACAACGC	CACTTCTGTGTATTCGGCCA
NOTCH2	CCCACAATGGACAGGACA	GAGGCGAAGGCACAATCA
NOTCH3	TGGTGTCCAGGGACGTCAG	GCAGATACCATGAGGGCCAC
HES1	AGTGAAGCACCTCCGGAAC	CGTTCATGCACTCGCTGA

Figure S2. Cisplatin-resistant hepatoblastoma cells have higher mRNA abundance of the stem cell marker, ALDH1A3, and the Notch signaling pathway. (**A**) Quantitative real-time PCR was utilized to assess the mRNA abundance of ALDH1A3, NOTCH1, NOTCH2, NOTCH3, and Hes1. Relative abundance of mRNA was calculated using the ΔΔCt method and reported as mean ± SEM of three biologic replicate experiments. Cisplatin-resistant cells from COA67 xenografts had significantly increased abundance of the stem cell marker ALDH1A3 and the NOTCH1 and NOTCH3 genes compared to naïve cells ($p<0.05$). (**B**) Sequences (5'-3') of forward and reverse primers used for real-time PCR (qPCR). Primers were designed using Primer3 web version 4.1.0 and checked for non-specific binding using the basic local alignment search tool (BLAST, NCBI). Notch1 primers were obtained from Applied Biosystems.

Figure S3

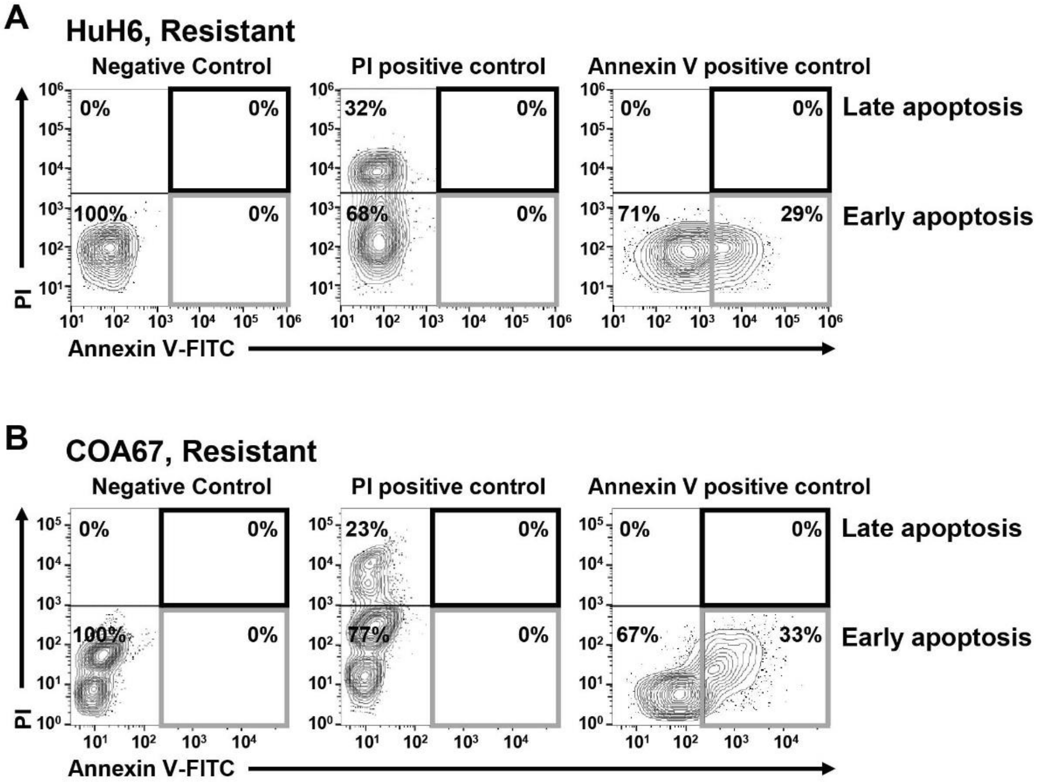

Figure S3. Representative contour plots of staining controls used in Annexin V/PI flow cytometry analysis. (**A**) HuH6 and (**B**) COA67 cisplatin-resistant cells were left unstained or stained with either Annexin V-FITC or PI alone and analyzed. Representative contour plots are shown. Early apoptosis is shown by the accumulation of Annexin V+ PI- cells (*lower right quadrant, grey boxes*) and late apoptosis is shown by the accumulation of Annexin V+ PI+ cells (*upper right quadrant, black boxes*).

Figure S4

A **HuH6**

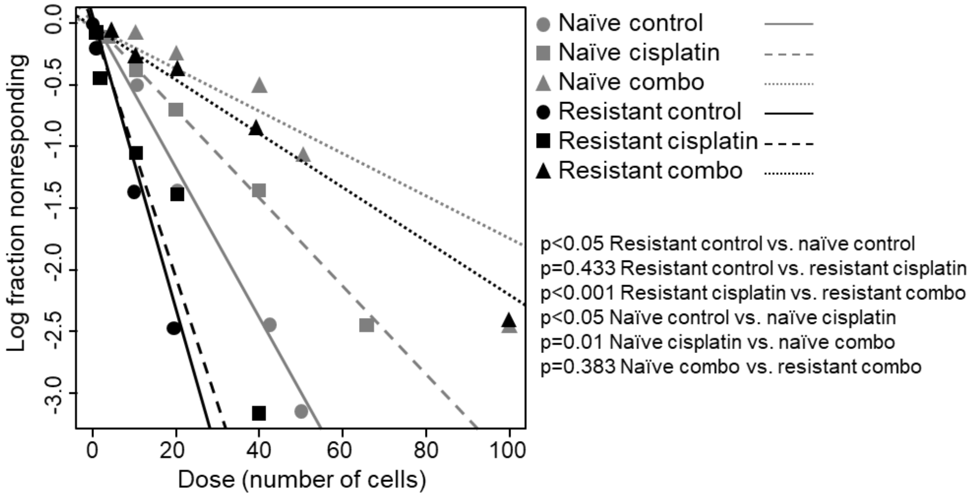

Group	1/(stem cell frequency) [95% CI]
**Naïve control**	15.53 [22.7, 10.6]
**Naïve cisplatin**	28.23 [40.4, 19.70]
**Naïve combination**	56.01 [82.3, 38.12]
**Resistant control**	7.18 [11.5, 4.49]
**Resistant cisplatin**	9.22 [14.2, 5.97]
**Resistant combination**	44.04 [63.7, 30.43]

B **COA67**

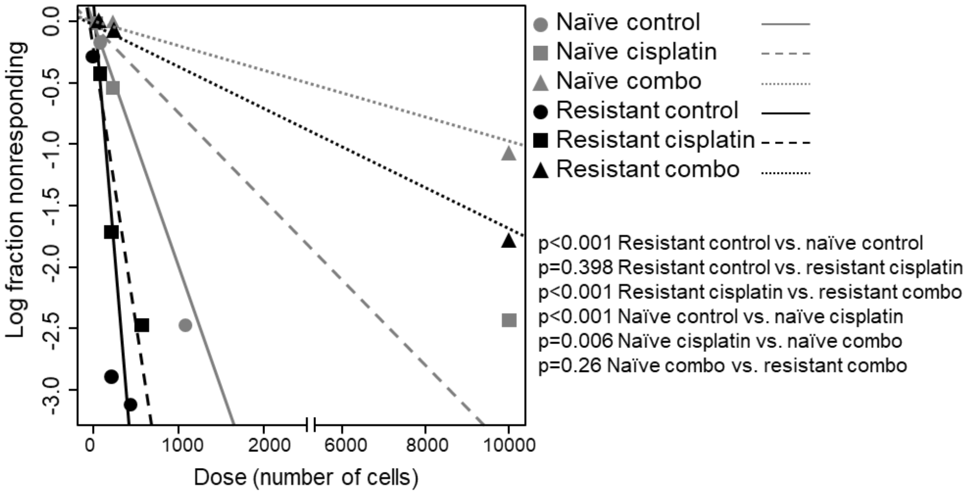

Group	1/(stem cell frequency) [95% CI]
**Naïve control**	446 [770, 257.7]
**Naïve cisplatin**	2820 [5809, 1368.8]
**Naïve combination**	10284 [20814, 5080.8]
**Resistant control**	128 [200, 82.5]
**Resistant cisplatin**	170 [269, 108.2]
**Resistant combination**	5901 [11270, 3089.5]

Figure S4. The addition of AZD1208 to cisplatin abrogates the increase in stem cell-like cancer cells (SCLCC) phenotype in cisplatin-resistant hepatoblastoma cells. Cisplatin-naïve and cisplatin-resistant (**A**) HuH6 and (**B**) COA67 cells were plated in non-adherent culture conditions at decreasing cell concentrations (from 5000 to 1 cell per well for HuH6 and 10000 to 1 cell per well for COA67) and treated with 10 μM cisplatin with and without the addition of 5 μM AZD1208. After 7 days, wells were examined by a single blinded researcher and the numbers of wells containing spheres were counted. Sphere forming ability was calculated utilizing the extreme limiting dilution software (http://bioinf.wehi.edu.au/software/elda/) and a plot of the log proportion of negative cultures vs. the number of cells plated is shown. The slope of the line is the estimated log-active SCLCC fraction. In both hepatoblastoma lines, cisplatin treatment did not affect sphere formation in resistant cells (*short dashed lines*, **A**, **B**), but decreased sphere formation in cisplatin-naïve cells (*short dashed lines*, **A**, **B**, $p<0.05$). Treatment with both AZD1208 and cisplatin significantly reduced sphere formation in both naïve and resistant HuH6 (*dotted lines*, **A**) and COA67 (*dotted lines*, **B**) cells compared to cisplatin alone, or to untreated controls ($p<0.05$). There was no difference between naïve and resistant cells treated with both AZD1208 and cisplatin, further demonstrating the ability of PIM inhibition to reduce sphere formation in resistant cells and abrogate the increase in SCLCC phenotype. Tables show estimated and 95% confidence intervals (CI, with lower and upper bounds shown in brackets) for the 1/(stem cell frequency) for each group corresponding to the ELDA plot above each table.

Figure S5

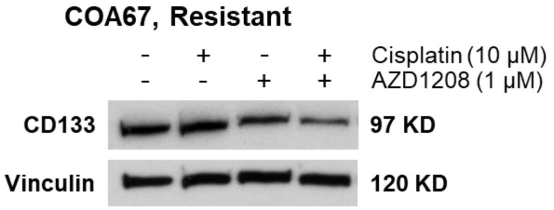

Figure S5. Treatment with both AZD1208 and cisplatin decreases CD133 protein expression in COA67 cisplatin-resistant cells. COA67 cisplatin-resistant cells were treated with cisplatin (at 10 μM), AZD1208 (at 1 μM), or both drugs for 72 hours and CD133 protein expression was evaluated by immunoblotting. Cisplatin treatment did not affect CD133 protein expression while treatment with AZD1208 decreased CD133 protein expression. Treatment with both AZD1208 and cisplatin significantly decreased CD133 protein expression in COA67 cisplatin-resistant cells compared to either cisplatin or AZD1208 alone, or to untreated control. Vinculin was used to confirm equal protein loading.

Figure S6

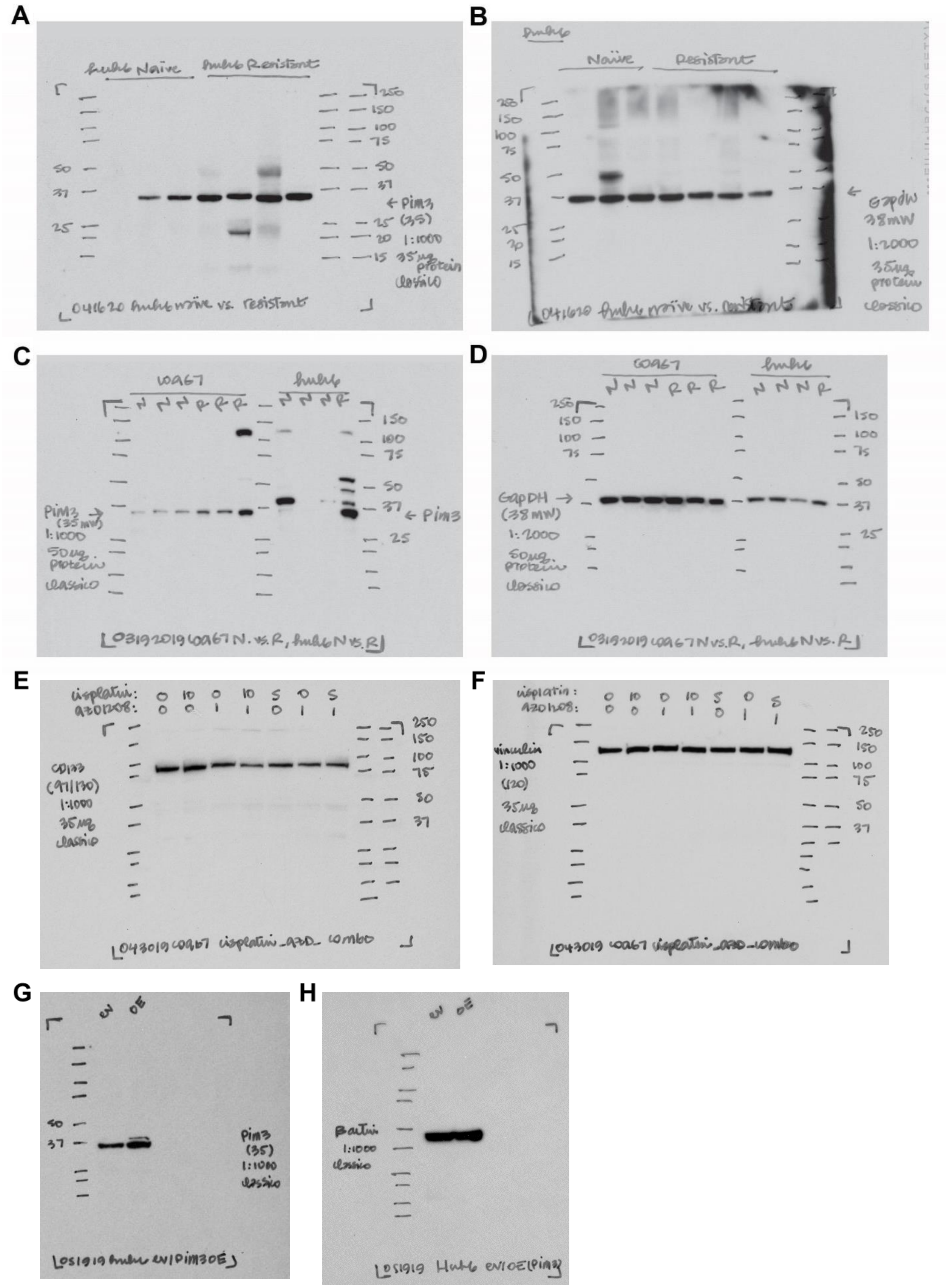

Figure S6. Full uncut Western blots are presented for the cropped Western blots shown in Figures 3 B, 7 A, and S5. (**A**) PIM3 and (**B**) GAPDH protein expression in the HuH6 cisplatin-naïve and cisplatin-resistant tumors. (**C**) PIM3 and (**D**) GAPDH protein expression in COA67 cisplatin-naïve and cisplatin-resistant tumors. (**E**) CD133 and (**F**) vinculin protein expression in COA67 cisplatin-resistant cells following treatment with cisplatin, AZD1208, or both drugs for 72 hours. (**G**) PIM3 and (**H**) β-actin protein expression in HuH6 empty vector (EV) and PIM3 overexpression (OE) cells.
